# Relationship of FDG PET/CT imaging features with tumor immune microenvironment and prognosis in colorectal cancer: a retrospective study

**DOI:** 10.1186/s40644-024-00698-4

**Published:** 2024-04-16

**Authors:** Jeong Won Lee, Hyein Ahn, Ik Dong Yoo, Sun-pyo Hong, Moo-Jun Baek, Dong Hyun Kang, Sang Mi Lee

**Affiliations:** 1 Department of Nuclear Medicine, Soonchunhyang University Cheonan Hospital, 31 Suncheonhyang 6-gil, Dongnam- gu, 31151 Cheonan, Korea; 2 Department of Pathology, CHA Gangnam Medical Center, CHA University School of Medicine, 569 Nonhyon-ro, Gangnam-gu, 06135 Seoul, Korea; 3 Department of Surgery, College of Medicine, Soonchunhyang University Cheonan Hospital, 31 Suncheonhyang 6- gil, Dongnam-gu, 31151 Cheonan, Korea; 4 Department of Colorectal surgery, College of Medicine, Soonchunhyang University Cheonan Hospital, 31 Suncheonhyang 6-gil, Dongnam-gu, 31151 Cheonan, Korea

**Keywords:** Colorectal cancer, F-18 fluorodeoxyglucose, Immune microenvironment, Positron emission tomography, Prognosis

## Abstract

**Background:**

Imaging features of colorectal cancers on 2-deoxy-2-[^18^F]fluoro-d-glucose (FDG) positron emission tomography/computed tomography (PET/CT) have been considered to be affected by tumor characteristics and tumor immune microenvironment. However, the relationship between PET/CT imaging features and immune reactions in tumor tissue has not yet been fully evaluated. This study investigated the association of FDG PET/CT imaging features in the tumor, bone marrow, and spleen with immunohistochemical results of cancer tissue and recurrence-free survival (RFS) in patients with colorectal cancer.

**Methods:**

A total of 119 patients with colorectal cancer who underwent FDG PET/CT for staging work-up and received curative surgical resection were retrospectively enrolled. From PET/CT images, 10 first-order imaging features of primary tumors, including intensity of FDG uptake, volumetric metabolic parameters, and metabolic heterogeneity parameters, as well as FDG uptake in the bone marrow and spleen were measured. The degrees of CD4+, CD8+, and CD163 + cell infiltration and interleukin-6 (IL-6) and matrix metalloproteinase-11 (MMP-11) expression were graded through immunohistochemical analysis of surgical specimens. The relationship between FDG PET/CT imaging features and immunohistochemical results was assessed, and prognostic significance of PET/CT imaging features in predicting RFS was evaluated.

**Results:**

Correlation analysis with immunohistochemistry findings showed that the degrees of CD4 + and CD163 + cell infiltration and IL-6 and MMP-11 expression were correlated with cancer imaging features on PET/CT. Patients with enhanced inflammatory response in cancer tissue demonstrated increased FDG uptake, volumetric metabolic parameters, and metabolic heterogeneity. FDG uptake in the bone marrow and spleen was positively correlated with the degree of CD163 + cell infiltration and IL-6 expression, respectively. In multivariate survival analysis, the coefficient of variation of FDG uptake in the tumor (*p* = 0.019; hazard ratio, 0.484 for 0.10 increase) and spleen-to-liver uptake ratio (*p* = 0.020; hazard ratio, 24.901 for 1.0 increase) were significant independent predictors of RFS.

**Conclusions:**

The metabolic heterogeneity of tumors and FDG uptake in the spleen were correlated with tumor immune microenvironment and showed prognostic significance in predicting RFS in patients with colorectal cancer.

**Supplementary Information:**

The online version contains supplementary material available at 10.1186/s40644-024-00698-4.

## Background

Colorectal cancer is the third most common cancer worldwide, and its prevalence is projected to increase by more than 60% in the next 20 years, with 1.9 million to 3.2 million new cases per year [1]. Radical surgical resection with or without neoadjuvant or adjuvant treatment is a potentially curative treatment for patients with colorectal cancer [1, 2]. However, even without distant metastatic lesions, up to 20% of patients with colorectal cancer experience cancer recurrence after curative surgery, necessitating the use of prognostic factors to predict clinical outcomes and aid in selecting optimal treatment strategies [3, 4].

Recently, interactions between cancer cells and host immune cells in the tumor microenvironment were found to significantly contribute to the development, progression, and treatment resistance of colorectal cancer [5–7]. Among immune cells, M2 type macrophages are one of the most notable cells that suppress immune responses and support tumor growth and invasion [5, 6]. M2 macrophages perform pro-tumoral functions by secreting diverse pro-inflammatory cytokines and enzymes, such as interleukin-6 (IL-6) and matrix metalloproteinase-11 (MMP-11) [8, 9]. In contrast, CD4 + and CD8 + T cells play a crucial role in anti-tumor immunity and help change the tumor microenvironment into a more tumor-suppressive condition [10, 11]. These diverse cells and components in the tumor immune microenvironment are considered potential treatment targets and prognostic factors for colorectal cancer [6].

In patients with colorectal cancer, 2-deoxy-2-[^18^F]fluoro-d-glucose (FDG) positron emission tomography/computed tomography (PET/CT) has shown a high diagnostic ability in case of distant metastasis of colorectal cancer, and has been recommended to assess ambiguous findings on conventional imaging examinations and to define the extent of metastatic disease [2, 12, 13]. Apart from its diagnostic role, PET/CT imaging features of colorectal cancer reflect the biological characteristics of the tumor and are significantly associated with clinical outcomes [14–16]. Furthermore, a previous study revealed that the degree of FDG uptake in the bone marrow (BM) and spleen, the major organs of the reticuloendothelial system, represented the degree of the host systemic inflammatory response and can be used to predict survival outcomes in patients with colorectal cancer [17, 18]. Because the host immune reaction in tumor tissue also affects FDG uptake in tumor lesions, FDG PET/CT is suggested as a non-invasive imaging modality to evaluate the tumor immune microenvironment [19, 20]. However, until now, only a few studies have investigated the relationship between FDG PET/CT imaging features and the condition of the immune microenvironment in colorectal cancer by histopathological analysis [19, 21].

This retrospective study investigated the relationship of FDG PET/CT imaging features of cancer lesions and the reticuloendothelial system with components of the tumor immune microenvironment using immunohistochemical analysis and recurrence-free survival (RFS) in patients with colorectal cancer treated with curative surgery.

## Methods

### Study participants

The electronic medical records of patients with histopathologically proven colorectal cancer at our medical center between January 2015 and December 2020 were retrospectively reviewed. Patients who met the following criteria were eligible for this study: patients who (1) underwent FDG PET/CT for the staging work-up of colorectal cancer, (2) received curative surgical resection including resection of synchronous hepatic metastasis, and (3) whose surgical specimens were available for immunohistochemical analysis. Patients who (1) were diagnosed with colorectal cancer other than adenocarcinoma, (2) had distant metastatic lesions other than hepatic metastasis, (3) had another malignant disease or acute inflammatory disease, (4) received neoadjuvant treatment or palliative surgery, (5) had inadequate surgical specimens for immunohistochemical analysis, or (6) were lost to follow-up within two years after surgery without events were excluded from the study. Based on the inclusion and exclusion criteria, 119 patients with colorectal cancer were enrolled in this study.

For staging work-up, preoperative blood tests including carcinoembryonic antigen (CEA) level, colonoscopy, chest and abdominopelvic CT, and FDG PET/CT were performed in all participants. After staging assessment, curative surgical resection, including resection of hepatic metastases, was performed. Based on the patients’ histopathological staging results and clinical condition, adjuvant treatment was administered. After treatments, routine follow-up assessments, including blood tests and imaging studies, were performed every 3–6 months.

### FDG PET/CT image analysis

The median interval between staging FDG PET/CT and surgery was 5 days (range, 2–20 days). FDG PET/CT was performed using the Biograph mCT 128 scanner (Siemens Healthineers, Knoxville, TN, USA). Before the PET/CT examination, the patients needed to be fasting for at least 6 h. After confirmation of a blood glucose level of < 200 mg/dl, FDG (approximately 4.07 MBq/kg) was intravenously injected. After 60 min of uptake time, PET/CT scanning was conducted from the skull base to the proximal thigh. Initially, non-contrast-enhanced CT data were acquired using automatic dose modulation with a reference current of 100 mA and 120 kVp (slice thickness 5 mm; slice increment 2.5 mm). PET data were then obtained for 1.5 min per bed position in the three-dimensional mode. PET images were reconstructed using the ordered-subset expectation maximization algorithm (21 subsets and two iterations). The matrix size and thickness of the PET images were 128 × 128 and 5 mm, respectively.

FDG PET/CT images of the enrolled patients were retrospectively reviewed based on the consensus of two board-certified nuclear medicine physicians using LIFEx software (version 7.3.0; www.lifexsoft.org) [22]. Reviewers could refer to images from conventional imaging examinations such as contrast-enhanced CT, but were blinded to other clinical information and outcomes. First, the imaging features of the primary colorectal cancer lesions were measured. Prior to computing the imaging features of the primary tumor, PET images of all patients were reconstructed into a voxel size of 4.07 × 4.07 × 2.5 mm. The primary tumor lesion was automatically delineated using Nestle’s adaptive threshold based on a previously published method [21, 23]: (standardized uptake value [SUV] threshold of tumor lesion) = 0.3 × (mean SUV of areas within tumor showing SUV of > 70% of maximum SUV of tumor) + (mean SUV of background). From PET images of the primary tumor, 10 imaging features were extracted: six conventional imaging features including maximum SUV, metabolic tumor volume (MTV), total lesion glycolysis (TLG), mean SUV, median SUV, and coefficient of variation of SUV (CoV SUV), and four imaging features from the SUV intensity histogram including kurtosis, skewness, entropy, and uniformity. Subsequently, FDG uptake in the BM, liver, and spleen was measured as described previously [18, 24]. To measure the FDG uptake in the BM, a spherical volume-of-interest (VOI) was drawn over six vertebral bodies of the thoracic and lumbar spines, excluding vertebrae with severe osteoarthritis, compression fractures, and post-operative changes. For each of the six VOIs, areas with SUV greater than 75% of the maximum SUV were automatically generated. The mean SUV of these areas was measured and defined as the BM SUV. To measure the FDG uptake in the liver and spleen, a spherical VOI of 3 cm was drawn in the right lobe of the liver while avoiding metastatic lesions, and a spherical VOI of 2 cm was drawn at the center of the spleen. The mean SUVs of these VOIs were calculated and defined as liver SUV and spleen SUV. The BM-to-liver uptake ratio (BLR) and spleen-to-liver uptake ratio (SLR) were calculated by dividing the BM SUV and the spleen SUV by the liver SUV.

### Immunohistochemical analysis

Two board-certified pathologists retrospectively performed an immunohistochemical analysis of the surgical specimens. Discrepancies between the pathologists during the analysis were resolved by consensus. The pathological stages of colorectal cancer were determined according to the American Joint Committee on Cancer staging guidelines eight edition, and the tumor grade was classified into two groups: low-grade tumors (well-differentiated and moderately-differentiated cancers) and high-grade tumors (poorly-differentiated, undifferentiated, and mucinous cancers). For the immunohistochemical analysis, cancer tissue areas that exhibited a characteristic cellular morphology without necrosis were selected. Immunohistochemical staining of CD4, CD8, CD163, IL-6, and MMP-11 was conducted using the following primary antibodies: monoclonal rabbit anti-human CD4 (clone SP35, catalog number: 7,904,423; Ventana Medical Systems, Tucson, AZ, USA), monoclonal mouse anti-human CD8 (clone C8/144B, catalog number: IR623; Dako, Carpinteria, CA, USA), monoclonal mouse anti-human CD163 (clone OTI2G12, catalog number: ab156769; Abcam, Cambridge, UK), polyclonal rabbit anti-human IL-6 (catalog number: ab6672; Abcam), and monoclonal rabbit anti-human MMP-11 (clone SN74-08, catalog number: NBP2-67670; Novus Biologicals, Centennial, CO, USA). Five representative areas were selected from the cancerous tissue for assessment using a high-power optical microscope at ×400 magnification. The numbers of infiltrating CD4+, CD8+, and CD163 + inflammatory cells in the cancer tissue were graded as follows: grade 0, ≤ 10 cells; grade 1, 11–50 cells; and grade 2 > 50 cells. The expression of IL-6 and MMP-11 in the tumor tissue was graded as follows: grade 0, negative; grade 1, weak expression (focal light brown color); and grade 2, moderate-to-intense expression (light-to-intense brown color).

### Statistical analysis

The Kruskal–Wallis test was performed to evaluate the relationship between FDG PET/CT imaging features and immunohistochemical findings in the tumor tissue. Post-hoc comparisons using Dunne’s test were conducted for PET/CT imaging parameters that showed statistical significance during the Kruskal–Wallis test. The Mann–Whitney U test was used to examine differences in PET/CT imaging features between low- and high-grade tumors. The prognostic significance of FDG PET/CT imaging features and clinical factors in predicting RFS was assessed using univariate and multivariate Cox proportional hazard regression analyses. RFS was defined as the duration from the date of surgery to the date of cancer recurrence detection, death, or the last follow-up visit. Variables that were found to be statistically significant in the univariate analysis were incorporated into the multivariate survival analysis. To estimate the cumulative survival curves, specific cut-off values were determined by receiver operating characteristic (ROC) curve analysis. Based on the cut-off values, the Kaplan–Meier method was applied to calculate cumulative RFS curves, and RFS curves between patient groups were compared using the log-rank test. MedCalc Statistical Software (version 22.016, MedCalc Software Ltd, Ostend, Belgium) was used for all statistical analyses. Statistical significance was set at *p* < 0.05.

## Results

### Patient characteristics

A total of 119 patients with colorectal cancer treated with curative surgery were selected as participants in this study, comprising 63 men (52.9%) and 56 women (47.1%) with a median age of 66 years (range, 35–84 years). Detailed information on the patient characteristics is summarized in Table 1. Of these patients, 8 (6.7%) had synchronous hepatic metastases. After surgery, 100 patients (84.0%) received adjuvant treatment.

**Table 1:** Baseline characteristics of the patients (*n* = 119)

Characteristics	Number of patients (%)
Age (years)	66 (35–84)*
Sex	
Men	63 (52.9%)
Women	56 (47.1%)
Tumor location	
Right colon	41 (34.5%)
Left colon	65 (54.6%)
Rectum	13 (10.9%)
Tumor size (cm)	5.1 (1.80–11.1)*
Tumor grade	
Low-grade	101 (74.9%)
High-grade	18 (15.1%)
Lymphovascular invasion	
Absent	76 (63.9%)
Present	43 (36.1%)
TNM stage	
Stage I	12 (10.1%)
Stage II	51 (42.9%)
Stage III	48 (40.3%)
Stage IV	8 (6.7%)
Serum CEA (ng/ml)	6.4 (1.2–625.2)*
CD4 + cell infiltration	
Grade 0	33 (27.7%)
Grade 1	41 (34.5%)
Grade 2	45 (37.8%)
CD8 + cell infiltration	
Grade 0	43 (36.1%)
Grade 1	44 (37.0%)
Grade 2	32 (26.9%)
CD163 + cell infiltration	
Grade 0	27 (22.7%)
Grade 1	46 (38.7%)
Grade 2	46 (38.7%)
IL-6 expression	
Grade 0	33 (27.7%)
Grade 1	38 (31.9%)
Grade 2	48 (40.3%)
MMP-11 expression	
Grade 0	45 (37.8%)
Grade 1	40 (33.6%)
Grade 2	34 (28.6%)
Adjuvant treatment	
Yes	100 (84.0%)
No	19 (16.0%)

*Median (range)

CEA, carcinoembryonic antigen; IL-6, interleukin-6; MMP-11, matrix metalloproteinase-11

### Correlation analysis between FDG PET/CT parameters and immunohistochemical results

**Fig. 1:**
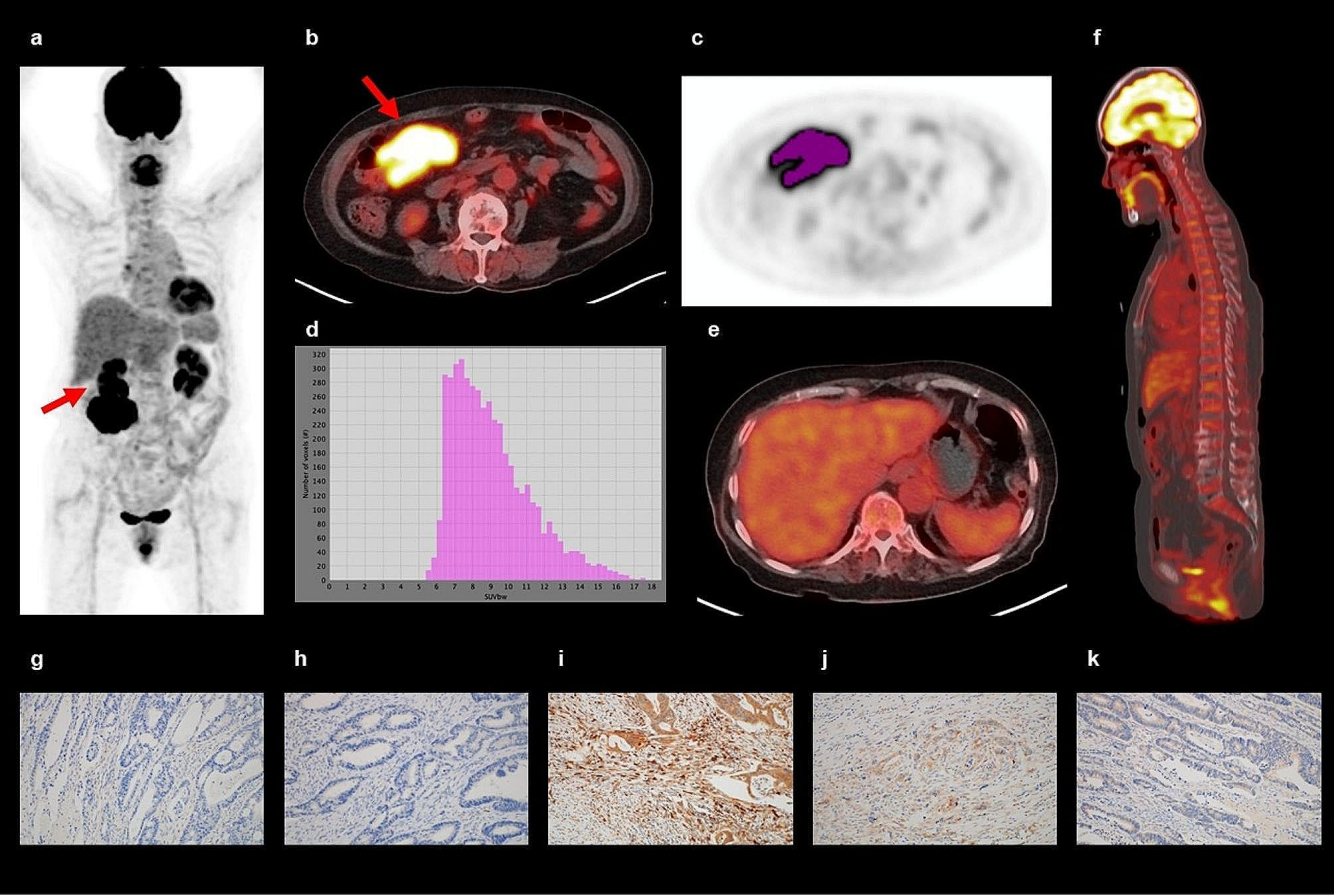
An 81-year-old woman underwent FDG PET/CT for staging work-up of colon cancer in the proximal transverse colon. In maximal intensity projection (**a**) and transaxial PET/CT (**b**) images, the tumor lesion (arrows) showed intensely increased FDG uptake with a maximum standardized uptake value (SUV) of 17.79. Using Nestle’s adaptive threshold method, the tumor lesion (purple color) was automatically delineated on PET images (**c**), and the SUV intensity histogram of the tumor lesion was made (**d**). Metabolic tumor volume, total lesion glycolysis, coefficient of variation, entropy, and uniformity extracted from the tumor lesion were 97.52 cm^3^, 1000.17 g, 0.233, 4.66, and 0.047, respectively. Increased FDG uptake of the bone marrow (BM) and spleen was observed in transaxial (**e**) and sagittal (**f**) PET/CT images, revealing BM SUV of 2.09, spleen SUV of 2.06, BLR of 1.00, and SLR of 0.98. The patient underwent curative surgery, and immunohistochemical analysis of the surgical specimen demonstrated grade 0 of CD4 + cell infiltration (**g**), grade 0 of CD8 + cell infiltration (**h**), grade 2 of CD163 + cell infiltration (**i**), grade 2 of interleukin-6 expression (**j**), and grade 1 of matrix metalloproteinase-11 expression (**k**) in tumor tissue. The patient experienced cancer recurrence 7 months after the surgery

The results of the correlation analysis between the FDG PET/CT imaging features and immunohistochemical findings are shown in Supplementary Tables 1–5. We further summarized the results of the correlation analysis, which showed statistical (*p* < 0.05) and borderline significance (*p* < 0.10), in Table 2 (Fig. 1).

**Fig. 2:**
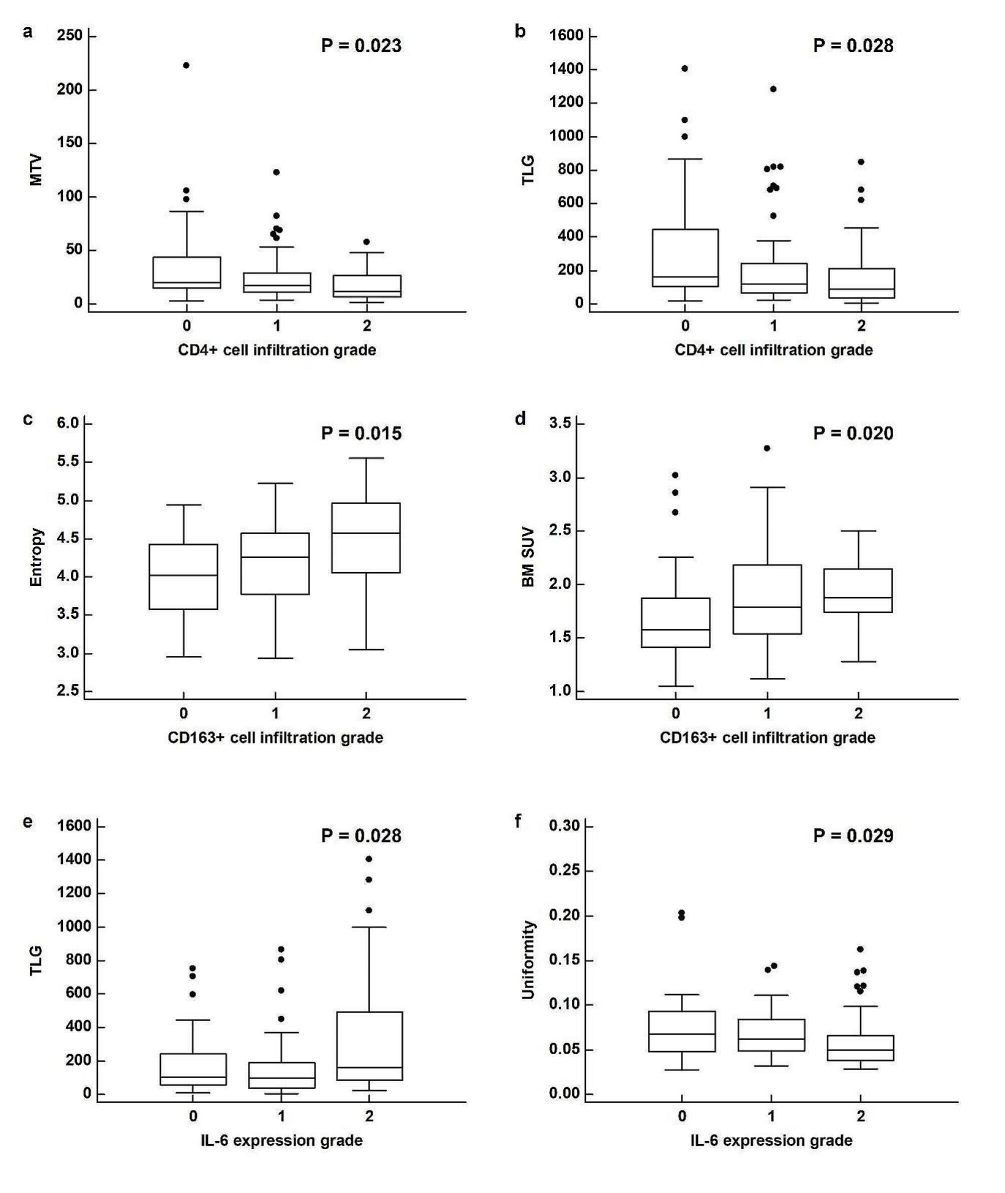
Distribution of metabolic tumor volume (MTV) (**a**) and total lesion glycolysis (TLG) (**b**) according to CD4 + cell infiltration grade in tumor tissue. Distribution of entropy (**c**) and mean standardized uptake value of the bone marrow (BM SUV) (**d**) according to CD163 + cell infiltration grade in tumor tissue. Distribution of TLG (**e**) and uniformity (**f**) according to interleukin-6 (IL-6) expression grade in tumor tissue

The CD4 + cell infiltration grade was significantly associated with MTV (*p* = 0.023) and TLG (*p* = 0.028), with the post-hoc analysis revealing significantly lower values in patients with grade 2 infiltration than in those with grade 0 infiltration (Fig. 2a and b). CD163 + cell infiltration was significantly correlated with entropy (*p* = 0.015) and BM SUV (*p* = 0.020), and showed a borderline significant correlation with CoV SUV and BLR (*p* < 0.10). Post-hoc analysis revealed that patients with grade 2 CD163 + cell infiltration had significantly higher entropy and BM SUV than those with grade 0 infiltration (*p* < 0.05; Fig. 2c and d). The CoV SUV tended to decrease as the CD163 + cell infiltration grade increased (*p* = 0.068), whereas BLR tended to increase as the grade increased (*p* = 0.067). IL-6 expression in tumor tissues was significantly correlated with TLG (*p* = 0.028) and uniformity (*p* = 0.029), and showed borderline significant correlations with maximum SUV, MTV, mean SUV, median SUV, spleen SUV, and SLR (*p* < 0.10). Post-hoc analysis revealed that the TLG value in patients with grade 2 IL-6 expression was significantly higher than that in patients with grades 0 and 1 IL-6 expression (Fig. 2e), and the uniformity value in patients with grade 2 IL-6 expression was significantly lower than that in patients with grade 0 IL-6 expression (*p* < 0.05; Fig. 2f). The spleen SUV (*p* = 0.077), SLR (*p* = 0.052), SUV (*p* = 0.097), MTV (*p* = 0.089), mean SUV (*p* = 0.078), and median SUV (*p* = 0.067) tended to increase as IL-6 expression grade increased. Regarding MMP-11, borderline significance was observed in the correlation with the CoV SUV (*p* = 0.066), showing a tendency to decrease as the MMP-11 expression grade increased. In contrast, CD8 + cell infiltration was not significantly correlated with any PET/CT imaging parameter (*p* > 0.05).

**Table 2:** Correlation analysis between FDG PET/CT imaging features and immunohistochemical results with p-value < 0.10

CD4 + cell	Grade 0	Grade 1	Grade 2	*P*-value
MTV (cm^3^)	20.25 (15.05–43.83)	16.99 (10.93–29.13)	11.60 (6.47–26.54)	0.023
TLG (g)	161.05 (105.23–442.98)	118.10 (66.49–243.70)	90.96 (36.61–212.22)	0.028
CD163 + cell	Grade 0	Grade 1	Grade 2	
CoV SUV	0.25 (0.22–0.33)	0.24 (0.20–0.27)	0.23 (0.21–0.29)	0.068
Entropy	4.02 (3.58–4.43)	4.26 (3.77–4.57)	4.57 (3.90–4.97)	0.015
BM SUV	1.62 (1.44–1.87)	1.79 (1.54–2.18)	1.84 (1.74–2.15)	0.020
BLR	0.77 (0.71–0.85)	0.81 (0.73–1.02)	0.87 (0.77–1.03)	0.067
IL-6	Grade 0	Grade 1	Grade 2	
Maximum SUV	12.38 (8.94–16.01)	12.21 (9.24–14.57)	13.35 (11.00–21.16)	0.097
MTV (cm^3^)	16.08 (6.81–24.87)	14.70 (6.73–27.70)	21.65 (11.60–37.94)	0.089
TLG (g)	101.89 (55.21–242.75)	100.98 (37.04–189.25)	161.17 (85.78–490.48)	0.028
Mean SUV	6.60 (5.26–9.33)	6.88 (5.44–8.18)	7.82 (6.23–10.26)	0.078
Median SUV	6.24 (5.15–8.90)	6.57 (5.17–8.22)	7.44 (6.09–10.13)	0.067
Uniformity	0.068 (0.048–0.093)	0.062 (0.049–0.085)	0.050 (0.038–0.066)	0.029
Spleen SUV	1.63 (1.49–1.89)	1.69 (1.55–1.88)	1.83 (1.62–2.01)	0.077
SLR	0.78 (0.71–0.87)	0.80 (0.76–0.84)	0.83 (0.78–0.96)	0.052
MMP-11	Grade 0	Grade 1	Grade 2	
CoV SUV	0.24 (0.20–0.32)	0.25 (0.23–0.30)	0.22 (0.19–0.30)	0.066

All values are expressed in median (interquartile range)

BLR, bone marrow-to-liver uptake ratio; BM, bone marrow; CoV, coefficient of variation; IL-6, interleukin-6; MMP-11, matrix metalloproteinase-11; MTV, metabolic tumor volume; SLR, spleen-to-liver uptake ratio; SUV, standardized uptake value; TLG, total lesion glycolysis

In addition to immunohistochemical analysis, we also performed a correlation analysis between tumor grade and FDG PET/CT imaging features (Supplementary Table 6). Patients with high-grade tumors showed significantly higher MTV (*p* = 0.008) and TLG (*p* = 0.006) values than those with low-grade tumors. Moreover, patients with high-grade tumors tended to exhibit a higher median SUV (*p* = 0.063) and a lower uniformity (*p* = 0.088) than those with low-grade tumors, with borderline statistical significance. Other PET/CT imaging features did not correlate with tumor grade (*p* > 0.10).

### Survival analysis for RFS

The patients’ clinical follow-up duration ranged from 26.1 to 84.4 months, with a median of 60.2 months. During follow-up, 30 patients (25.2%) had recurrence or death events, and the 5-year RFS rate was 73.8% (95% confidence interval [CI], 65.5–82.1%). The prognostic relevance of clinical factors and FDG PET/CT imaging features was assessed using univariate and multivariate survival analysis. In the univariate analysis, TNM stage (*p* = 0.015), CoV SUV (*p* = 0.031), uniformity (*p* = 0.050), BLR (*p* = 0.044), and SLR (*p* = 0.005) were significantly associated with RFS (Table 3). The maximum SUV revealed only borderline significance (*p* = 0.075), and neither MTV nor TLG exhibited statistical significance (*p* > 0.05). Five variables that demonstrated a significant association with RFS in the univariate analysis were included in the multivariate analysis (Table 3). Multivariate analysis showed that TNM stage (*p* = 0.031; hazard ratio, 2.341; 95% CI, 1.079–5.078 for stage III-IV), CoV SUV (*p* = 0.019; hazard ratio, 0.484; 95% CI, 0.263–0.889 for 0.10 increase), and SLR (*p* = 0.020; hazard ratio, 24.901; 95% CI, 1.660–373.709 for 1.0 increase) remained significant independent prognostic factors for RFS. Uniformity and BLR failed to show statistical significance in the multivariate analysis (*p* > 0.05).

**Table 3:** Univariate and multivariate survival analysis for recurrence-free survival

Variables		Univariate analysis		Multivariate analysis	
		*P*-value	Hazard ratio (95% CI)	*P*-value	Hazard ratio (95% CI)
Age	(1-year increase)	0.756	1.005 (0.973–1.038)		
Sex	Woman	Reference	1.000		
	Man	0.544	0.801 (0.391–1.640)		
Tumor location	Left colon	Reference	1.000		
	Right colon	0.480	1.320 (0.611–2.855)		
	Rectum	0.578	1.371 (0.451–4.167)		
Serum CEA level	< 5 ng/ml	Reference	1.000		
	≥ 5 ng/ml	0.088	1.869 (0.912–3.828)		
Tumor size	(1 cm increase)	0.102	1.077 (0.880–1.354)		
Tumor grade	Low-grade	Reference	1.000		
	High-grade	0.777	1.149 (0.439–3.005)		
Lymphovascular invasion	Absent	Reference	1.000		
	Present	0.080	1.388 (0.938–2.338)		
TNM stage	Stage I-II	Reference	1.000	Reference	1.000
	Stage III-IV	0.015	2.554 (1.195–5.460)	0.031	2.341 (1.079–5.078)
Adjuvant therapy	No	Reference	1.000		
	Yes	0.201	0.556 (0.226–1.367)		
Maximum SUV	(1.0 increase)	0.075	1.041 (0.980–1.102)		
MTV	(10.0 cm^3^ increase)	0.425	1.054 (0.926–1.200)		
TLG	(10.0 g increase)	0.617	0.999 (0.986–1.013)		
Mean SUV	(1.0 increase)	0.185	0.884 (0.767–1.017)		
Median SUV	(1.0 increase)	0.089	1.009 (0.974–1.045)		
CoV SUV	(0.10 increase)	0.031	0.503 (0.269–0.942)	0.019	0.484 (0.263–0.889)
Kurtosis	(1.0 increase)	0.930	1.018 (0.682–1.521)		
Skewness	(1.0 increase)	0.811	1.151 (0.365–3.624)		
Entropy	(1.0 increase)	0.433	1.277 (0.693–2.530)		
Uniformity	(0.10 increase)	0.050	0.416 (0.173–1.000)	0.664	0.984 (0.290–3.653)
BM SUV	(1.0 increase)	0.221	1.646 (0.742–3.652)		
BLR	(1.0 increase)	0.044	5.128 (1.030–23.282)	0.261	2.870 (0.456–18.049)
Spleen SUV	(1.0 increase)	0.293	1.766 (0.612–5.098)		
SLR	(1.0 increase)	0.005	45.21 (3.212–636.387)	0.020	24.901 (1.660–373.709)

BLR, bone marrow-to-liver uptake ratio; BM, bone marrow; CEA, carcinoembryonic antigen; CI, confidence interval; CoV, coefficient of variation; MTV, metabolic tumor volume; SLR, spleen-to-liver uptake ratio; SUV, standardized uptake value; TLG, total lesion glycolysis

**Fig. 3:**
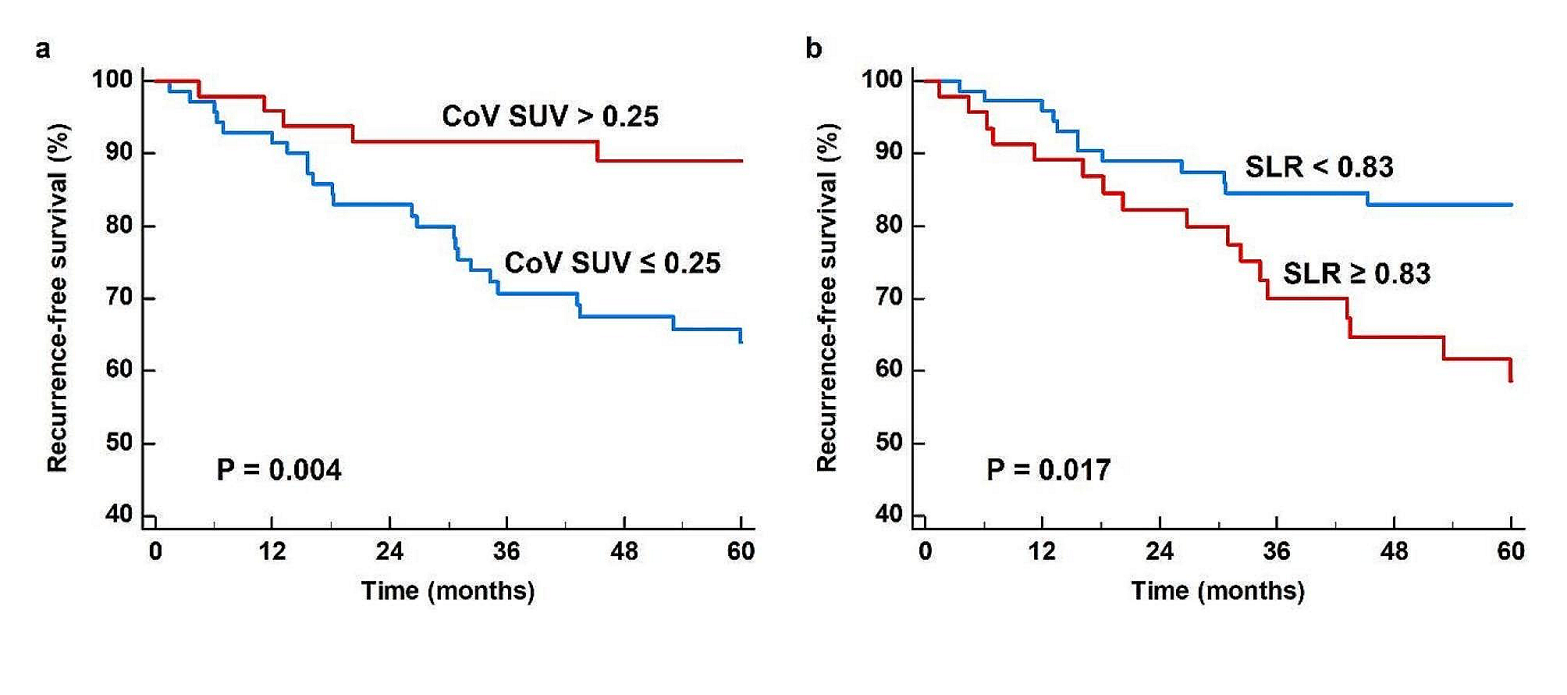
Kaplan-Meier curves of recurrence-free survival according to coefficient of variation of tumor SUV (CoV SUV) (**a**) and spleen-to-liver uptake ratio (SLR) (**b**)

To estimate the cumulative survival curves, the enrolled patients were classified into two groups according to the specific cut-off values of the CoV SUV (0.25) and SLR (0.83), as determined by ROC curve analysis. Patients with low CoV SUV and high SLR showed significantly worse RFS than those with high CoV SUV (*p* = 0.004) and low SLR (*p* = 0.017) (Fig. 3). The 5-year RFS rates in patients with low CoV SUV and high SLR were 63.9% (95% CI, 52.2–75.6%) and 58.6% (95% CI, 43.2–74.0%), respectively, while the 5-year RFS rates in patients with high CoV SUV and low SLR were 89.0% (95% CI, 79.4–98.1%) and 82.9% (95% CI, 74.1–91.7%), respectively.

The patients’ recurrence rates were further compared based on the combination of TNM stage, CoV SUV, and SLR (Table 4). Patients with high CoV SUV and low SLR had low recurrence rates of 5.3% (stage I-II) and 6.7% (stage III-IV), respectively, according to the TNM stage. In contrast, TNM stage III-IV patients with low CoV SUV and high SLR in PET/CT images showed a high recurrence rate of 65.0%. Even among patients with stage I-II disease, the recurrence rate was as high as 33.3% if the patients showed both a low CoV SUV and high SLR.

**Table 4:** Recurrence rates according to the combination of TNM stage, CoV SUV, and SLR

		TNM stage	
		Stage I-II	Stage III-IV
CoV SUV and SLR	CoV SUV > 0.25 and SLR < 0.83	1/19 (5.3%)	1/15 (6.7%)
	CoV SUV ≤ 0.25 or SLR ≥ 0.83	5/32 (15.6%)	6/21 (28.6%)
	CoV SUV ≤ 0.25 and SLR ≥ 0.83	4/12 (33.3%)	13/20 (65.0%)

CoV, coefficient of variation; SLR, spleen-to-liver uptake ratio; SUV, standardized uptake value

## Discussion

In the present study, we measured the maximum SUV, volumetric parameters, and other first-order imaging features of colorectal cancer lesions in FDG PET/CT images, and assessed their relationship with immunohistochemistry findings in the tumor immune microenvironment. The maximum SUV, which represents the highest value of FDG uptake in a tumor, is the most widely used PET/CT parameter for estimating tumor FDG uptake [25]. However, unlike in studies on other malignant diseases, the maximum SUV failed to show a significant association with tumor aggressiveness and prognosis in several studies of colorectal cancer, suggesting that other factors, such as inflammatory conditions in the tumor microenvironment, could affect the maximum SUV of colorectal cancers [14, 21, 26]. MTV and TLG are the most notable volumetric metabolic parameters and are known to reflect the metabolic burden of cancerous lesions more accurately than the maximum SUV [27]. The CoV SUV is the ratio of the standard deviation of the SUV to the mean SUV, which reflects heterogeneity of FDG uptake [16]. The four SUV intensity histogram parameters, kurtosis, skewness, entropy, and uniformity, represent the shape, asymmetry, randomness, and homogeneity of the SUV distribution in the SUV intensity histogram [20]. A lesion that shows high entropy and low uniformity in the histogram is considered to have increased metabolic heterogeneity [20, 28].

Among the components of the tumor immune microenvironment, the degrees of CD4 + cell and CD163 + cell infiltration and IL-6 expression in cancer tissues were significantly correlated with FDG PET/CT imaging features of primary tumors. Although CD4 + T cells can polarize into different subsets, which either stimulate or inhibit the immune response to cancer cells, higher levels of CD4 + cell infiltration in tumor tissues were related to better clinical outcomes in colorectal cancer in previous studies [10, 11, 29]. In our study, MTV and TLG showed significant negative correlations with CD4 + cell infiltration and significant positive correlations with tumor grade. This finding suggests that colorectal cancers with CD4 + T cell depletion may have a high metabolic tumor burden and aggressive features. Macrophages are the most abundant immune cell type in the tumor microenvironment, and M2 type macrophages exhibit high levels of CD163 expression [6]. M2 macrophages are also the most important source of IL-6 secretion in the tumor microenvironment [30]. IL-6 derived from M2 type macrophages activates the Janus kinase 2/signal transducer activator of the transcription 3 pathway, promoting angiogenesis and inflammation in tumor tissues and enhancing the invasion, proliferation, and survival of cancer cells [6, 8, 30]. Therefore, high levels of M2 macrophage infiltration and IL-6 expression in tumor tissues are significantly associated with poor prognosis in patients with colorectal cancer [6, 8]. Correlation analysis in this study revealed that the degree of CD163 + cell infiltration was positively correlated with the metabolic heterogeneity of tumor lesions, and increased IL-6 expression was observed in tumor lesions with increased FDG uptake, volumetric parameters, and metabolic heterogeneity. Our results indicated that tumors with severe pro-tumoral immune reactions showed increased FDG uptake, volumetric parameters, and metabolic heterogeneity. Until now, the relationship between PET/CT imaging features and the inflammatory response in the tumor immune microenvironment has not been reported. Our study demonstrated that PET/CT imaging parameters of tumor lesions might serve as imaging biomarkers for evaluating the status of the tumor immune microenvironment in colorectal cancer. However, considering the significant overlap of the PET/CT imaging features between grades of immunohistochemical findings, FDG PET/CT findings alone might be limited for assessing the immune microenvironment, and further research is need to validate our results. Currently, immunotherapy is considered ineffective in most patients with colorectal cancer, except for in those with high microsatellite instability or mismatch repair deficiency [31]. On the other hand, many trials targeting the immune microenvironment to treat colorectal cancer have been performed [6, 31]. In future clinical trials, FDG PET/CT imaging features of tumor lesions might provide clinical significance in selecting optimal candidates and predicting treatment responses.

MMP-11 expression as well as CD163 + cell infiltration were negatively correlated with the CoV SUV, with borderline statistical significance. MMP-11 belongs to the zinc-dependent endopeptidases family, and is expressed in colorectal cancer cells and macrophages in tumor tissues [9, 32]. MMP-11 is involved in extracellular matrix remodeling and contributes to tumor growth, invasion, and metastasis [32]. Regarding the clinical value of the CoV SUV, conflicting results have been shown in other types of cancers; however, in previous studies of colorectal cancer, patients with a high CoV SUV had a better response to chemoradiotherapy and a lower risk of recurrence than those with low values [16, 33]. Considering the results of our study, it could be speculated that the high CoV SUV in colorectal cancer lesions on FDG PET/CT represented an unfavorable tumor microenvironment for tumor growth.

In addition to primary cancer lesion imaging features, we also evaluated the relationship of FDG uptake of the BM and spleen with immunohistochemistry results. FDG uptake values in both the BM and spleen are known to be imaging biomarkers of the systemic inflammatory response to cancer cells, but both values are considered to reflect different aspects of the host’s immune response [18, 24]. In previous studies that evaluated the relationship of FDG uptake in the BM and spleen with immunohistochemical findings in cervical and gastric cancer specimens, FDG uptake in the BM was positively associated with the degree of myeloid-derived suppressor cell and CD163 + macrophage infiltration in the tumor tissue [24, 34]. Meanwhile, FDG uptake in the spleen was positively correlated with the degree of CD8 + T cell lymphocyte, CD20 + B cell lymphocyte, and CD68 + monocyte infiltration in the tumor tissue [24, 35]. Furthermore, a previous study on patients with biliary tract and pancreatic cancer patients reported high serum levels of various cytokines, including IL-6, in those with high spleen FDG uptake [36]. In colorectal cancer, only two studies have investigated the prognostic significance of FDG uptake in the BM and spleen, and its relationship with histopathological findings in cancer tissues has not been evaluated [17, 18]. Similar to the results of the previous studies, those of the present study revealed increased FDG uptake in the BM and spleen was observed in colorectal cancers with increased CD163 + cell infiltration and IL-6 expression, suggesting an intense inflammatory response in the tumor tissue of patients with high FDG uptake in the BM and spleen. These results further supported the utilization of FDG uptake in the BM and spleen as imaging biomarkers for estimating the host inflammatory response in colorectal cancer [17, 18].

Among the FDG PET/CT imaging features, CoV SUV and SLR were determined to be independent prognostic factors for predicting RFS in the multivariate survival analysis, along with the TNM stage. Intratumoral metabolic heterogeneity and FDG uptake in the spleen, rather than the maximum SUV, volumetric parameters, and FDG uptake in the BM, were significantly associated with the clinical outcomes of patients with colorectal cancer. Patients with low CoV SUV and high SLR had significantly worse RFS than those with high CoV SUV and low SLR, demonstrating 5-year RFS rates of only 63.9% and 58.6% in those with low CoV SUV and high SLR, respectively. Moreover, our results showed that combining these two PET/CT imaging features with the TNM stage could further stratify the risk of recurrence after surgery. Patients with both high CoV SUV and low SLR had recurrence rates of less than 10%, irrespective of the TNM stage. Meanwhile, up to 65% of stage III-IV patients with both low CoV SUV and high SLR experienced recurrence. Considering the relationship between the CoV SUV and SLR with the tumor immune microenvironment, our results could be an imaging evidence demonstrating that the inflammatory response in the tumor microenvironment significantly impacts clinical outcomes. Among patients with advanced colorectal cancer, those who show findings indicative of an enhanced inflammatory response on staging work-up FDG PET/CT should undergo intensive management with careful surveillance.

In the present study, we used modified Nestle’s adaptive threshold method for delineating colorectal cancer lesions. Since the tumor segmentation method could affect the values of tumor imaging features on FDG PET/CT, numerous tumor segmentation methods have been proposed such as the threshold-based and algorithm-based methods [37]. Nestle’s method was originally developed to delineate target volume in radiotherapy planning [23]. It is one of the threshold-based methods that used the threshold calculated from FDG uptake in the tumor and background on a case-by-case basis, and has demonstrated strong inter-patient stability in a previous study that compared PET segmentation methods [37, 38]. In several previous studies, the clinical value of tumor PET radiomic parameters were successfully demonstrated using Nestle’s method, suggesting the clinical relevance of this method [20, 39]. However, because each segmentation method has its advantages and disadvantages, it could be beneficial to select different segmentation methods according to the purpose of the study [37]. Further studies are needed to systematically compare segmentation methods and to reach a consensus on selecting an optimal method for use in radiomic studies involving FDG PET/CT.

The current study had several limitations. First, this study was retrospectively performed at a single-center with a limited number of subjects. Therefore, the results require further validation. Second, we excluded patients who received neoadjuvant treatment and included only those whose surgical specimens were available for immunohistochemical analysis. This might have led to selection bias in this study. Third, the CoV SUV, one of the independent prognostic factors in our study, is known to be affected by the SUV threshold used for tumor delineation [16]. Different SUV thresholds may produce different values of mean and standard deviation SUV in tumor lesions, which has been suggested as one of the reasons for the contradictory results related to the clinical value of CoV SUV in previous studies [16, 40]. Therefore, further research is needed to establish the optimal SUV threshold for calculating the CoV SUV in colorectal cancer lesions. Finally, the mechanisms underlying the relationship between FDG PET/CT imaging features and immunohistochemistry results should be further investigated in future studies.

## Conclusion

FDG PET/CT imaging features of primary tumor lesions and the reticuloendothelial system revealed a significant association with the tumor immune microenvironment and RFS in patients with colorectal cancer. CD4 + T cell infiltration in tumor tissues was negatively correlated with metabolic volumetric parameters. Patients with severe CD163 + macrophage infiltration and increased IL-6 expression showed increased FDG uptake, metabolic volumetric parameters, and metabolic heterogeneity of tumor lesions, as well as increased FDG uptake in the BM and spleen. In the multivariate survival analysis, the CoV SUV and SLR were independent prognostic factors for predicting RFS. The metabolic heterogeneity of the tumors and FDG uptake in the spleen might serve as potential imaging biomarkers for evaluating the immune response in the tumor microenvironment and for predicting recurrence risk after curative surgery in patients with colorectal cancer.

### Electronic supplementary material

Below is the link to the electronic supplementary material.

Supplementary Material 1

## Data Availability

The datasets used and/or analysed during the current study are available from the corresponding author on reasonable request.

## Abbreviations

BM: Bone marrow
BLR: Bone marrow-to-liver uptake ratio
CEA: Carcinoembryonic antigen
COV: Coefficient of variation
FDG: 2-deoxy-2-[^18^F]fluoro-d-glucose
IL-6: Interleukin-6
MMP-11: Matrix metalloproteinase-11
MTV: Metabolic tumor volume
PET/CT: Positron emission tomography/computed tomography
RFS: Recurrence-free survival
ROC: Receiver operating characteristic
SUV: Standardized uptake value
SLR: Spleen-to-liver uptake ratio
TLG: Total lesion glycolysis
VOI: Volume-of-interest
