# Supplementary material for: Relationship of FDG PET/CT imaging features with tumor immune microenvironment and prognosis in colorectal cancer: a retrospective study
Source: Cancer Imaging. 2024 Apr 16;24:53. doi: 10.1186/s40644-024-00698-4 (PMC11020988; doi:10.1186/s40644-024-00698-4)
Supplement: Supplementary file 1 — Supplementary Material 1 [file 40644_2024_698_MOESM1_ESM.docx]

**Supplementary Table 1**. Results of correlation analysis between FDG PET/CT imaging features and CD4+ cell infiltration grade in tumor tissue.

|  | Grade 0 | Grade 1 | Grade 2 | P-value |
| --- | --- | --- | --- | --- |
| Maximum SUV | 14.14 (11.39–18.57) | 12.25 (9.49–16.98) | 11.95 (7.84–16.76) | 0.291 |
| MTV | 20.25 (15.05–43.83) | 16.99 (10.93–29.13) | 11.60 (6.47–26.54) | 0.023 |
| TLG | 161.05 (105.23–442.98) | 118.10 (66.49–243.70) | 90.96 (36.61–212.22) | 0.028 |
| Mean SUV | 7.79 (6.62–9.32) | 7.06 (5.94–8.48) | 6.83 (5.19–9.06) | 0.280 |
| Median SUV | 7.46 (6.46–8.83) | 6.59 (5.58–8.17) | 6.43 (5.04–8.91) | 0.250 |
| CoV SUV | 0.24 (0.20–0.29) | 0.24 (0.22–0.31) | 0.23 (0.20–0.34) | 0.965 |
| Kurtosis | -0.46 (-0.72–0.14) | -0.52 (-0.76–-0.05) | -0.54 (-0.81–-0.26) | 0.509 |
| Skewness | 0.56 (0.47–0.78) | 0.62 (0.41–0.76) | 0.56 (0.43–0.69) | 0.762 |
| Entropy | 4.37 (3.84–4.55) | 4.31 (4.02–4.71) | 4.12 (3.59–4.74) | 0.580 |
| Uniformity | 0.054 (0.043–0.070) | 0.061 (0.044–0.079) | 0.066 (0.046–0.094) | 0.416 |
| BM SUV | 1.75 (1.52–2.10) | 1.77 (1.54–2.21) | 1.75 (1.55–1.90) | 0.752 |
| BLR | 0.79 (0.72–0.97) | 0.81 (0.73–0.94) | 0.78 (0.73–0.94) | 0.996 |
| Spleen SUV | 1.72 (1.59–2.03) | 1.71 (1.56–1.90) | 1.72 (1.50–1.88) | 0.304 |
| SLR | 0.84 (0.77–0.97) | 0.81 (0.73–0.88) | 0.80 (0.77–0.86) | 0.244 |

All values are expressed in median (interquartile range).

BLR, bone marrow-to-liver uptake ratio; BM, bone marrow; CoV, coefficient of variation; MTV, metabolic tumor volume; SLR, spleen-to-liver uptake ratio; SUV, standardized uptake value; TLG, total lesion glycolysis

**Supplementary Table 2**. Results of correlation analysis between FDG PET/CT imaging features and CD8+ cell infiltration grade in tumor tissue.

|  | Grade 0 | Grade 1 | Grade 2 | P-value |
| --- | --- | --- | --- | --- |
| Maximum SUV | 12.81 (9.31–16.37) | 12.32 (7.84–17.75) | 13.35 (10.91–17.42) | 0.677 |
| MTV | 16.99 (9.13–27.64) | 16.10 (8.29–36.17) | 19.50 (8.64–37.95) | 0.865 |
| TLG | 121.78 (57.48–213.18) | 110.01 (45.89–404.26) | 135.44 (63.83–308.47) | 0.735 |
| Mean SUV | 7.06 (5.95–8.71) | 7.01 (5.17–9.48) | 7.35 (6.18–9.05) | 0.748 |
| Median SUV | 6.80 (5.75–8.35) | 6.75 (4.93–9.48) | 6.95 (6.04–8.73) | 0.773 |
| CoV SUV | 0.23 (0.20–0.29) | 0.24 (0.20–0.30) | 0.25 (0.22–0.33) | 0.299 |
| Kurtosis | -0.46 (-0.76–-0.08) | -0.40 (-0.82–0.02) | -0.54 (-0.73–-0.31) | 0.876 |
| Skewness | 0.60 (0.45–0.73) | 0.60 (0.44–0.78) | 0.56 (0.36–0.68) | 0.734 |
| Entropy | 4.31 (3.85–4.70) | 4.21 (3.54–4.65) | 4.39 (4.01–4.85) | 0.443 |
| Uniformity | 0.055 (0.042–0.083) | 0.064 (0.048–0.098) | 0.053 (0.042–0.070) | 0.284 |
| BM SUV | 1.74 (1.53–1.88) | 1.69 (1.46–2.05) | 1.84 (1.62–2.19) | 0.168 |
| BLR | 0.77 (0.71–0.92) | 0.78 (0.70–0.90) | 0.84 (0.75–1.03) | 0.149 |
| Spleen SUV | 1.68 (1.51–1.81) | 1.79 (1.58–1.93) | 1.84 (1.56–1.94) | 0.191 |
| SLR | 0.79 (0.75–0.88) | 0.83 (0.76–0.92) | 0.81 (0.79–0.88) | 0.440 |

All values are expressed in median (interquartile range).

BLR, bone marrow-to-liver uptake ratio; BM, bone marrow; CoV, coefficient of variation; MTV, metabolic tumor volume; SLR, spleen-to-liver uptake ratio; SUV, standardized uptake value; TLG, total lesion glycolysis

**Supplementary Table 3**. Results of correlation analysis between FDG PET/CT imaging features and CD163+ cell infiltration grade in tumor tissue.

|  | Grade 0 | Grade 1 | Grade 2 | P-value |
| --- | --- | --- | --- | --- |
| Maximum SUV | 12.71 (9.84–18.12) | 12.21 (8.76–14.55) | 13.40 (9.43–19.68) | 0.512 |
| MTV | 17.82 (10.36–37.62) | 15.87 (7.70–24.54) | 18.98 (9.39–37.22) | 0.504 |
| TLG | 111.98 (65.14–419.54) | 117.81 (46.66–173.41) | 150.27 (62.16–377.18) | 0.505 |
| Mean SUV | 7.40 (5.74–9.69) | 6.76 (5.28–8.24) | 7.65 (6.14–9.24) | 0.300 |
| Median SUV | 6.96 (5.36–9.36) | 6.41 (5.17–7.77) | 7.29 (5.90–9.38) | 0.283 |
| CoV SUV | 0.25 (0.22–0.33) | 0.24 (0.20–0.27) | 0.23 (0.21–0.29) | 0.068 |
| Kurtosis | -0.46 (-0.71–-0.21) | -0.38 (-0.72–-0.13) | -0.55 (-0.81–-0.26) | 0.287 |
| Skewness | 0.53 (0.48–0.78) | 0.61 (0.41–0.77) | 0.56 (0.41–0.66) | 0.497 |
| Entropy | 4.02 (3.58–4.43) | 4.26 (3.77–4.57) | 4.57 (3.90–4.97) | 0.015 |
| Uniformity | 0.055 (0.043–0.071) | 0.064 (0.049–0.090) | 0.053 (0.042–0.077) | 0.416 |
| BM SUV | 1.62 (1.44–1.87) | 1.79 (1.54–2.18) | 1.84 (1.74–2.15) | 0.020 |
| BLR | 0.77 (0.71–0.85) | 0.81 (0.73–1.02) | 0.87 (0.77–1.03) | 0.067 |
| Spleen SUV | 1.68 (1.53–1.84) | 1.70 (1.52–1.90) | 1.79 (1.56–1.96) | 0.412 |
| SLR | 0.78 (0.74–0.84) | 0.81 (0.76–0.88) | 0.82 (0.77–0.91) | 0.464 |

All values are expressed in median (interquartile range).

BLR, bone marrow-to-liver uptake ratio; BM, bone marrow; CoV, coefficient of variation; MTV, metabolic tumor volume; SLR, spleen-to-liver uptake ratio; SUV, standardized uptake value; TLG, total lesion glycolysis

**Supplementary Table 4**. Results of correlation analysis between FDG PET/CT imaging features and IL-6 expression grade in tumor tissue.

|  | Grade 0 | Grade 1 | Grade 2 | P-value |
| --- | --- | --- | --- | --- |
| Maximum SUV | 12.38 (8.94–16.01) | 12.21 (9.24–14.57) | 13.35 (11.00–21.16) | 0.097 |
| MTV | 16.08 (6.81–24.87) | 14.70 (6.73–27.70) | 21.65 (11.60–37.94) | 0.089 |
| TLG | 101.89 (55.21–242.75) | 100.98 (37.04–189.25) | 161.17 (85.78–490.48) | 0.028 |
| Mean SUV | 6.60 (5.26–9.33) | 6.88 (5.44–8.18) | 7.82 (6.23–10.26) | 0.078 |
| Median SUV | 6.24 (5.15–8.90) | 6.57 (5.17–8.22) | 7.44 (6.09–10.13) | 0.067 |
| CoV SUV | 0.23 (0.19–0.30) | 0.24 (0.21–0.28) | 0.24 (0.22–0.32) | 0.629 |
| Kurtosis | -0.54 (-0.85–-0.04) | -0.46 (-0.72–0.07) | -0.51 (-0.75–-0.22) | 0.775 |
| Skewness | 0.59 (0.39–0.67) | 0.63 (0.49–0.77) | 0.56 (0.41–0.71) | 0.291 |
| Entropy | 4.15 (3.80–4.69) | 4.24 (3.89–4.58) | 4.37 (3.89–4.757) | 0.835 |
| Uniformity | 0.068 (0.048–0.093) | 0.062 (0.049–0.085) | 0.050 (0.038–0.066) | 0.029 |
| BM SUV | 1.68 (1.41–2.15) | 1.76 (1.56–1.90) | 1.83 (1.57–2.10) | 0.550 |
| BLR | 0.77 (0.69–1.00) | 0.79 (0.74–0.92) | 0.84 (0.72–0.95) | 0.656 |
| Spleen SUV | 1.63 (1.49–1.89) | 1.69 (1.55–1.88) | 1.83 (1.62–2.01) | 0.077 |
| SLR | 0.78 (0.71–0.87) | 0.80 (0.76–0.84) | 0.83 (0.78–0.96) | 0.052 |

All values are expressed in median (interquartile range).

BLR, bone marrow-to-liver uptake ratio; BM, bone marrow; CoV, coefficient of variation; IL-6, interleukin-6; MTV, metabolic tumor volume; SLR, spleen-to-liver uptake ratio; SUV, standardized uptake value; TLG, total lesion glycolysis

**Supplementary Table 5**. Results of correlation analysis between FDG PET/CT imaging features and MMP-11 expression grade in tumor tissue.

|  | Grade 0 | Grade 1 | Grade 2 | P-value |
| --- | --- | --- | --- | --- |
| Maximum SUV | 12.65 (9.77–18.27) | 13.68 (10.45–16.86) | 11.46 (7.66–15.50) | 0.464 |
| MTV | 17.82 (10.59–35.77) | 16.33 (6.43–27.68) | 16.26 (9.39–26.15) | 0.449 |
| TLG | 133.90 (71.49–327.73) | 114.87 (48.46–213.57) | 110.95 (56.62–224.35) | 0.504 |
| Mean SUV | 7.36 (6.00–9.02) | 7.41 (6.17–9.24) | 6.85 (5.18–9.05) | 0.646 |
| Median SUV | 6.93 (5.71–8.74) | 6.95 (5.75–9.09) | 6.60 (4.97–8.66) | 0.725 |
| CoV SUV | 0.24 (0.20–0.32) | 0.25 (0.23–0.30) | 0.22 (0.19–0.30) | 0.066 |
| Kurtosis | -0.40 (-0.74–-0.13) | -0.51 (-0.81–0.01) | -0.51 (-0.74–-0.27) | 0.845 |
| Skewness | 0.60 (0.45–0.72) | 0.60 (0.44–0.77) | 0.52 (0.41–0.72) | 0.604 |
| Entropy | 4.12 (3.87–4.63) | 4.44 (3.96–4.66) | 4.38 (3.65–4.80) | 0.513 |
| Uniformity | 0.056 (0.041–0.077) | 0.054 (0.046–0.080) | 0.066 (0.044–0.101) | 0.500 |
| BM SUV | 1.74 (1.48–2.07) | 1.77 (1.54–2.07) | 1.84 (1.62–2.14) | 0.604 |
| BLR | 0.78 (0.71–1.02) | 0.80 (0.72–0.91) | 0.83 (0.77–0.96) | 0.707 |
| Spleen SUV | 1.77 (1.56–1.89) | 1.66 (1.51–1.92) | 1.72 (1.58–2.02) | 0.425 |
| SLR | 0.81 (0.76–0.92) | 0.80 (0.75–0.84) | 0.83 (0.77–0.89) | 0.338 |

All values are expressed in median (interquartile range).

BLR, bone marrow-to-liver uptake ratio; BM, bone marrow; CoV, coefficient of variation; MMP-11, matrix metalloproteinase-11; MTV, metabolic tumor volume; SLR, spleen-to-liver uptake ratio; SUV, standardized uptake value; TLG, total lesion glycolysis

**Supplementary Table 6**. Results of correlation analysis between FDG PET/CT images features and grade of colorectal cancer.

|  | Low-grade | High-grade | P-value |
| --- | --- | --- | --- |
| Maximum SUV | 12.38 (9.33–16.01) | 15.97 (10.51–21.78) | 0.186 |
| MTV | 15.04 (7.62–27.75) | 27.63 (17.05–47.97) | 0.008 |
| TLG | 110.20 (51.15–204.38) | 218.70 (126.82–679.63) | 0.006 |
| Mean SUV | 6.95 (5.61–8.92) | 8.39 (6.78–10.58) | 0.120 |
| Median SUV | 6.66 (5.28–8.35) | 8.05 (6.66–9.85) | 0.063 |
| CoV SUV | 0.24 (0.21–0.32) | 0.24 (0.20–0.27) | 0.563 |
| Kurtosis | -0.52 (-0.80–-0.16) | -0.36 (-0.67–0.12) | 0.140 |
| Skewness | 0.57 (0.44–0.71) | 0.64 (0.41–0.80) | 0.436 |
| Entropy | 4.30 (3.86–4.66) | 4.33 (3.74–4.71) | 0.778 |
| Uniformity | 0.062 (0.045–0.086) | 0.050 (0.038–0.062) | 0.088 |
| BM SUV | 1.75 (1.51–1.96) | 1.98 (1.67–2.22) | 0.144 |
| BLR | 0.79 (0.71–0.92) | 0.89 (0.77–1.00) | 0.269 |
| Spleen SUV | 1.71 (1.54–1.92) | 1.82 (1.63–2.00) | 0.483 |
| SLR | 0.80 (0.76–0.88) | 0.82 (0.77–0.98) | 0.558 |

All values are expressed in median (interquartile range).

BLR, bone marrow-to-liver uptake ratio; BM, bone marrow; CoV, coefficient of variation; MTV, metabolic tumor volume; SLR, spleen-to-liver uptake ratio; SUV, standardized uptake value; TLG, total lesion glycolysis
